# *Pseudomonas aeruginosa* Enhances Production of a Non-Alginate Exopolysaccharide during Long-Term Colonization of the Cystic Fibrosis Lung

**DOI:** 10.1371/journal.pone.0082621

**Published:** 2013-12-06

**Authors:** Holly K. Huse, Taejoon Kwon, James E. A. Zlosnik, David P. Speert, Edward M. Marcotte, Marvin Whiteley

**Affiliations:** 1 Section of Molecular Genetics and Microbiology, University of Texas at Austin, Austin, Texas, United States of America; 2 Institute of Cellular and Molecular Biology and Center for Systems and Synthetic Biology, University of Texas at Austin, Austin, Texas, United States of America; 3 Department of Pediatrics and Center for Understanding and Preventing Infection in Children, The University of British Columbia, Vancouver, British Columbia, Canada; East Carolina University School of Medicine, United States of America

## Abstract

The gram-negative opportunistic pathogen *Pseudomonas aeruginosa* is the primary cause of chronic respiratory infections in individuals with the heritable disease cystic fibrosis (CF). These infections can last for decades, during which time *P. aeruginosa* has been proposed to acquire beneficial traits via adaptive evolution. Because CF lacks an animal model that can acquire chronic *P. aeruginosa* infections, identifying genes important for long-term *in vivo* fitness remains difficult. However, since clonal, chronological samples can be obtained from chronically infected individuals, traits undergoing adaptive evolution can be identified. Recently we identified 24 *P. aeruginosa* gene expression traits undergoing parallel evolution *in vivo* in multiple individuals, suggesting they are beneficial to the bacterium. The goal of this study was to determine if these genes impact *P. aeruginosa* phenotypes important for survival in the CF lung. By using a gain-of-function genetic screen, we found that 4 genes and 2 operons undergoing parallel evolution *in vivo* promote *P. aeruginosa* biofilm formation. These genes/operons promote biofilm formation by increasing levels of the non-alginate exopolysaccharide Psl. One of these genes, *phaF*, enhances Psl production via a post-transcriptional mechanism, while the other 5 genes/operons do not act on either *psl* transcription or translation. Together, these data demonstrate that *P. aeruginosa* has evolved at least two pathways to over-produce a non-alginate exopolysaccharide during long-term colonization of the CF lung. More broadly, this approach allowed us to attribute a biological significance to genes with unknown function, demonstrating the power of using evolution as a guide for targeted genetic studies.

## Introduction

Chronic infections are an especially difficult healthcare problem because pathogens persist in the host despite therapeutic treatment. Due to a genetic defect, individuals with the heritable disease cystic fibrosis (CF) are prone to chronic, fatal respiratory infections caused by the Gram-negative opportunistic pathogen *Pseudomonas aeruginosa* [1]. During chronic infection, *P. aeruginosa* undergoes adaptation in the CF lung, which is thought to enhance the fitness of this bacterium *in vivo* [2,3]. Therefore, identifying fitness traits in chronic CF strains is important for designing novel treatment strategies, but this problem is challenging because animal models that recapitulate chronic *P. aeruginosa* CF infections do not currently exist. 

One means of overcoming this challenge is to utilize an evolutionary approach to identify beneficial adaptations. Chronic CF infections are well suited for evolutionary studies because they are typically dominated by a single *P. aeruginosa* strain allowing clonal, chronological isolates to be sampled over decades-long time periods [4]. Beneficial adaptations can be determined by 1) identifying parallel genotypes and phenotypes that arise within multiple *P. aeruginosa* lineages, a strong indicator of adaptive evolution and 2) using whole-genome sequencing to identify evidence for positive selection. Indeed, phenotypic studies have shown that *P. aeruginosa* frequently undergoes parallel changes *in vivo*, one of the most well understood being conversion to the mucoid phenotype [5]. This phenotype is characterized by over-production of the exopolysaccharide alginate, which enhances biofilm formation [6]. Prevalence of mucoid isolates is on average reported as ~41% [4,7,8], though one study reported 80% [9]. These data suggest that other important adaptive traits likely arise *in vivo*. Additionally, genome sequencing has demonstrated adaptation occurring during the first 40,000 of up to 200,000 generations *in vivo* [2,3]. This initial period of adaptation is followed by a longer period of genetic drift and negative selection [3], suggesting that adaptive traits are likely to arise during initial phases (~40,000 generations) of chronic infection.

We previously used an evolutionary approach to identify *P. aeruginosa* gene expression traits likely undergoing adaptive evolution *in vivo* [10]. *P. aeruginosa* was chronologically sampled from 3 CF patients, ranging from the first infecting bacterium (the ancestor) to ~40,000 generations post-infection. By comparing gene expression profiles of early and late isolates sampled from multiple patients, we identified 24 parallel gene expression changes that occurred over time within each lineage. These data strongly indicate that these gene expression traits are undergoing adaptive evolution and therefore benefit the bacterium during chronic infection.

Of these 24 genes, 15 were up-regulated in chronic isolates compared to their ancestor; however, the phenotypic consequences associated with these gene expression changes were unclear. Several of these genes were previously associated with biofilm formation, a sessile mode of growth prominent in the CF lung presumably due to its protective effects against antibiotics and host defenses [11-14]. We therefore hypothesized that a portion of these genes would promote enhanced biofilm formation. To test this hypothesis, we performed a gain-of-function screen to determine the impact of these up-regulated genes on biofilm formation. Our results reveal that expression of 4 genes and 2 operons increases biofilm formation via enhancing levels of the P. *aeruginosa* exopolysaccharide Psl. One of the genes, the transcriptional regulator *phaF*, enhances Psl levels via a post-transcriptional mechanism, while the other genes enhance Psl production but do not affect either *psl* transcription or translation. Finally, we show that within the first ~40,000 generations of chronic infection, Psl production increases in ~72% of chronic CF isolates compared to their ancestor. These data indicate that enhanced production of Psl is an important adaptation during the first ~40,000 generations of *P. aeruginosa* growth in the CF lung and that multiple pathways evolved to impact this trait.

## Materials and Methods

### Bacterial strains and growth media

The bacterial strains used in this study are listed in Tables S3 and S4. *Escherichia coli* was routinely grown on Luria-Bertani medium at 37°C. *P. aeruginosa* strains were grown at 37°C on tryptic soy broth/agar medium, morpholinepropanesulfonic acid (MOPS)-buffered medium (50 mM MOPS [pH 7.2], 93 mM NH_4_Cl, 43 mM NaCl, 3.7 mM KH_2_PO_4_, 1 mM MgSO_4_, and 3.5 μM FeSO_4_7H_2_O) supplemented with 0.5% glucose and 0.5% casamino acids, or a previously described Synthetic Cystic Fibrosis Medium (SCFM) [15]. When applicable, antibiotics were used at the following concentrations: 10 μg/ml and 25 μg/ml gentamicin for maintenance and selection, respectively, in *E. coli* and 50 μg/ml and 100 μg/ml gentamicin for maintenance and selection, respectively, in *P. aeruginosa*.

### DNA and plasmid manipulations

The primers used in this study are listed in Table S5. Standard methods were used for DNA and plasmid manipulations.

### Strain construction

A non-polar deletion of *pslA* in PAO1 was constructed using allelic replacement and *sacB* counter-selection as previously described [16]. *pslA-lacZ* transcriptional and translational fusions were a generous gift from Dr. Yasuhiko Irie and were introduced onto the chromosome at the *attB* site as previously described [17]. Allelic replacement and chromosomal integrations were confirmed via PCR analyses and DNA sequencing.

### Microtiter dish biofilm assay

Microtiter dish biofilm assays were performed as previously described with minor modifications [18]. Strains were grown in MOPS minimal media supplemented with 0.5% glucose and 0.5% casamino acids. Overnight *P. aeruginosa* cultures were diluted to OD_600_=1.0 in MOPS minimal medium, and 5 μl was used to inoculate 95 μl media (final OD_600_=0.05) in a 96-well polystyrene microtiter dish (NUNC). Each well contained a 3 mm borosilicate glass bead for aeration. Microtiter dishes were sealed with parafilm and incubated at 37°C with 250 r.p.m. shaking. Staining with 0.1% crystal violet was performed at 12 h, followed by washing and dye solubilization with 33% acetic acid. Absorbance was measured at 620 nm. Strains were grown in octuplicate and at least 3 biological replicates were performed. 

### Microarray analyses

The empty vector control strain and the *phaF* over-expression strain were grown in 250 ml flasks in 25 ml MOPS minimal media supplemented with 0.5% glucose and 0.5% casamino acids. RNA was prepared from cells grown to OD_600_=0.9-1.0. Clinical isolates and PAO1 were grown in SCFM and RNA was prepared from cells grown to OD_600_=0.4-0.5 [10]. RNA extraction, cDNA synthesis, hybridization, and downstream data analyses were performed as previously described [10,15]. Genes were considered differentially expressed if they exhibited a greater than 2-fold change and a false discovery rate (FDR) < 0.05.

### Psl immunoblots

Psl immunoblots were carried out as previously described with minor modifications [19]. Stationary phase cultures were diluted to a final OD_600_=~0.05 in 5 ml MOPS minimal media supplemented with 0.5% glucose and 0.5% casamino acids or SCFM. Cultures were grown for approximately 16 h, and either 10 OD equivalents (volume [ml] = 10/culture OD_600_) or whole cultures were harvested. Cell pellets were re-suspended in 100 μl of 0.5 M EDTA, boiled at 100°C for 30 minutes, and centrifuged. The supernatant fraction was treated with proteinase K (final concentration 5 mg/ml) for 1 h at 60°C, followed by inactivation for 30 minutes at 80°C. Samples were stored at 4°C for Psl immunoblotting. Pelleted lysate was re-suspended in 1 ml 6 M urea and boiled for 1 h at 100°C. Protein concentration was measured via Bradford assay (Bio-Rad). Crude polysaccharide preparations were normalized to equal protein concentrations by diluting samples in 0.5 M EDTA. 20-30 μl of polysaccharide preparation was spotted onto a nitrocellulose membrane using a dot blot apparatus. Blocking was performed in 10% skim milk in TBST (20 mM Tris, 137 mM NaCl, 0.1% Tween 20, pH 7.6) followed by probing with α-Psl antiserum (1:25,000 in TBST) for 45 minutes at 25°C with agitation. After washing, the membrane was incubated with horseradish peroxidase (HRP)-conjugated goat anti-rabbit secondary antibody (1:10,000 in TBST; Bio-Rad) for 1 h at room temperature. The membrane was washed and developed using SuperSignal West Dura Extended Duration Substrate following the manufacturer’s instructions (Pierce). At least 3 biological replicates were performed for each strain.

### β-galactosidase assays

*P. aeruginosa* strains were grown as described above. 1 ml culture aliquots were removed at exponential (OD_600_=0.5-1.0) and stationary phase (~16 hours) for β-galactosidase measurements. Cells were pelleted, re-suspended in 1 ml Z-buffer + 2-mercaptoethanol, lysed with 200 μl chloroform, and equilibrated for 5 minutes. β-galactosidase activity was measured using the Galacto-Light Plus System (Applied Biosystems) and normalized to total protein as measured by Bradford assay.

### RNA preparation and Northern blot analyses

*P. aeruginosa* PAO1 strains were grown as described above and cultures were mixed 1:1 (vol/vol) with RNAlater (Ambion). Total RNA was purified using RNAbee following the manufacturer’s protocol (Tel-Test). RNA samples were treated with DNase to remove contaminating DNA following the manufacturer’s protocol. Probe sequences are listed in Table S5. Northern blot analyses were performed as previously described [20]. 

### Microarray data accession numbers

All microarray data are available at the NCBI GEO database. The accession number for the microarrays comparing PAO1 and ancestor clinical isolates is GSE21966. The accession number for the microarrays comparing the empty vector control strain and the *phaF* over-expression strain is GSE47173.

## Results

### Over-expression of up-regulated genes enhances biofilm formation

**Figure 1:**
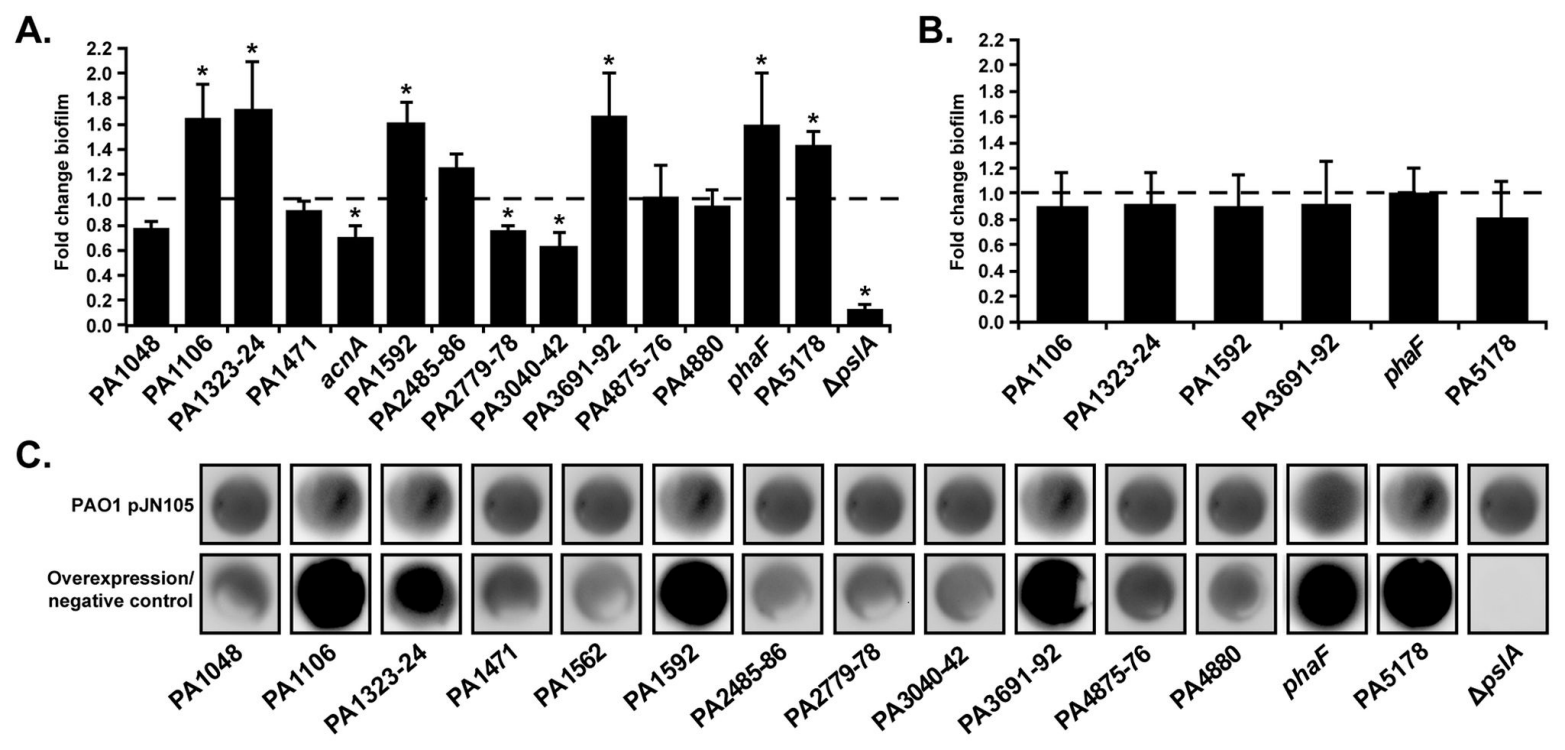
Biofilm formation and Psl production in over-expression strains. (A) Fold change in biofilm formation of wild-type PAO1 over-expression strains. Bars represent the average fold change in biofilm formation compared to the empty vector control strain, PAO1 pJN105 (dashed line). Δ*pslA* pJN105, a strain that cannot produce Psl, served as a negative control. While fold change in biofilm formation is reported for clarity, statistics were performed on absorbance values for empty vector control and over-expression strains assayed on the same day. At least 3 biological replicates were performed in octuplicate. *, *P* value < 0.001 by Student’s *t* test. Error bars represent standard error of the mean, n=3. (B) Fold change in biofilm formation of Δ*pslA* over-expression strains. Bars represent the average fold change in biofilm formation compared to the empty vector control strain, Δ*pslA* pJN105 (dashed line). Statistics and error bars are reported as in A. (C) Psl production in PAO1 pJN105 (top) and PAO1 over-expression strains (bottom). The top panels represent Psl production in the wild-type empty vector control strain, PAO1 pJN105, while the bottom panels represent Psl production in the over-expression strains, or as in the last bottom panel, the negative control strain Δ*pslA* pJN105. At least 3 biological replicates were performed.

Recently we identified 24 *P. aeruginosa* gene expression traits undergoing selection within the CF lung [10]. Fifteen of the 24 genes were up-regulated in chronic isolates compared to the ancestor (defined as the P. *aeruginosa* isolate initially establishing the chronic infection), and their roles during chronic infection are unknown. Some of these genes are predicted to be part of an operon and have been associated with biofilm formation in previous studies [11,13,14]. To test the hypothesis that some of these up-regulated genes/operons contribute to biofilm formation, we used a genetic approach to test for gain-of-function phenotypes. Each gene or operon was introduced into the laboratory strain *P. aeruginosa* PAO1 on a low-copy plasmid and expressed from the arabinose-inducible pBAD promoter [21]. This genetic system is relevant for studying gain-of-function phenotypes because 1) the pBAD promoter exhibits minimal expression without arabinose inducer [21], allowing us to avoid non-specific cellular responses caused by gene over-expression; 2) the PAO1 genome is sequenced and pathways impacting biofilm formation are well characterized; and 3) microarray analyses showed that expression of the 15 up-regulated genes are comparable between PAO1 and ancestral clinical isolates (Table S1). We conducted our experiments without arabinose since this resulted in an approximate 3-4 fold increase in expression, levels similar to those found in chronic clinical isolates (Table S2). Expression of PA1106, PA1323-24, PA1592, PA3691-92, *phaF* (PA5060), and PA5178 enhanced biofilm formation relative to the empty vector control strain (Figure 1A). While the phenotype is modest, similar increases in biofilm formation have been reported [22]. As expected, a negative control strain unable to initiate attachment, PAO1 Δ*pslA* pJN105, showed a significant biofilm defect (Figure 1A). Among the genes and operons that enhanced biofilm formation, only PA3692 (*lptF*) and *phaF* have known or proposed functions. PA3692 is involved in adhesion to lung epithelial cells, while *phaF* is a transcriptional regulator of polyhydroxyalkanoate biosynthesis in *Pseudomonas putida* [23,24]. The remaining biofilm-enhancing genes have hypothetical functions. 

### Enhanced biofilm formation of *P. aeruginosa* requires Psl

We next sought to determine the mechanism governing enhanced biofilm formation. Although multiple pathways are important for *P. aeruginosa* biofilm formation, we obtained an important clue to the mechanism when one of the adapted genes (*phaF*) was over-expressed in the P. *aeruginosa* laboratory strain PA14. Surprisingly, this strain did not produce more biofilm (Figure S1). Examination of the PAO1 and PA14 genomes revealed that PA14 does not encode the genetic machinery required for production of the exopolysaccharide Psl. Since Psl is important for biofilm formation in *P. aeruginosa*, we hypothesized that enhanced biofilm formation of PAO1 over-expressing *phaF*, and potentially the other adapted genes, involves Psl production. To test this hypothesis, we expressed the adapted genes/operons in a PAO1 strain that lacks the ability to produce Psl (Δ*pslA*) and tested these strains for biofilm formation (Figure 1B). As in PA14, *phaF* expression did not enhance biofilm formation in Δ*pslA*. Notably, the other 5 strains also did not form more biofilm than the empty vector control strain, Δ*pslA* pJN105 (Figure 1B), suggesting that these 6 genes/operons enhance biofilm formation in a Psl-dependent manner.

### Up-regulated genes induce Psl production

If Psl is required for enhanced biofilm formation in the over-expression strains, each should correspondingly produce more Psl. We therefore used immunoblot analyses to measure Psl production in all 14 of our over-expression strains. First, Psl anti-sera specificity was demonstrated with a strain that cannot produce Psl (WFPA800) [19], an arabinose-inducible *psl* over-expression strain (WFPA801) [19], and the wild-type empty vector control strain PAO1 pJN105 (Figure S2). To determine the effect of each gene/operon on Psl production, crude polysaccharide extracts from each over-expression strain were subjected to anti-Psl immunoblot analyses. PAO1 Δ*pslA* carrying the empty vector served as the negative control, and as expected, Psl was not detected (Figure 1C). As predicted from our biofilm results, over-expression strains PA1106, PA1323-24, PA1592, PA3691-92, *phaF*, and PA5178 produced more Psl than the empty vector control strain (Figure 1C). Importantly, increased Psl is not an artificial response to gene over-expression, as 8 over-expression strains did not show enhanced biofilm formation or Psl production. These data along with the fact that enhanced biofilm formation does not occur in PAO1 Δ*pslA* suggests that these 6 genes/operons enhance biofilm formation through increased production of the exopolysaccharide Psl. 

### *phaF* regulates Psl production post-transcriptionally

**Figure 2:**
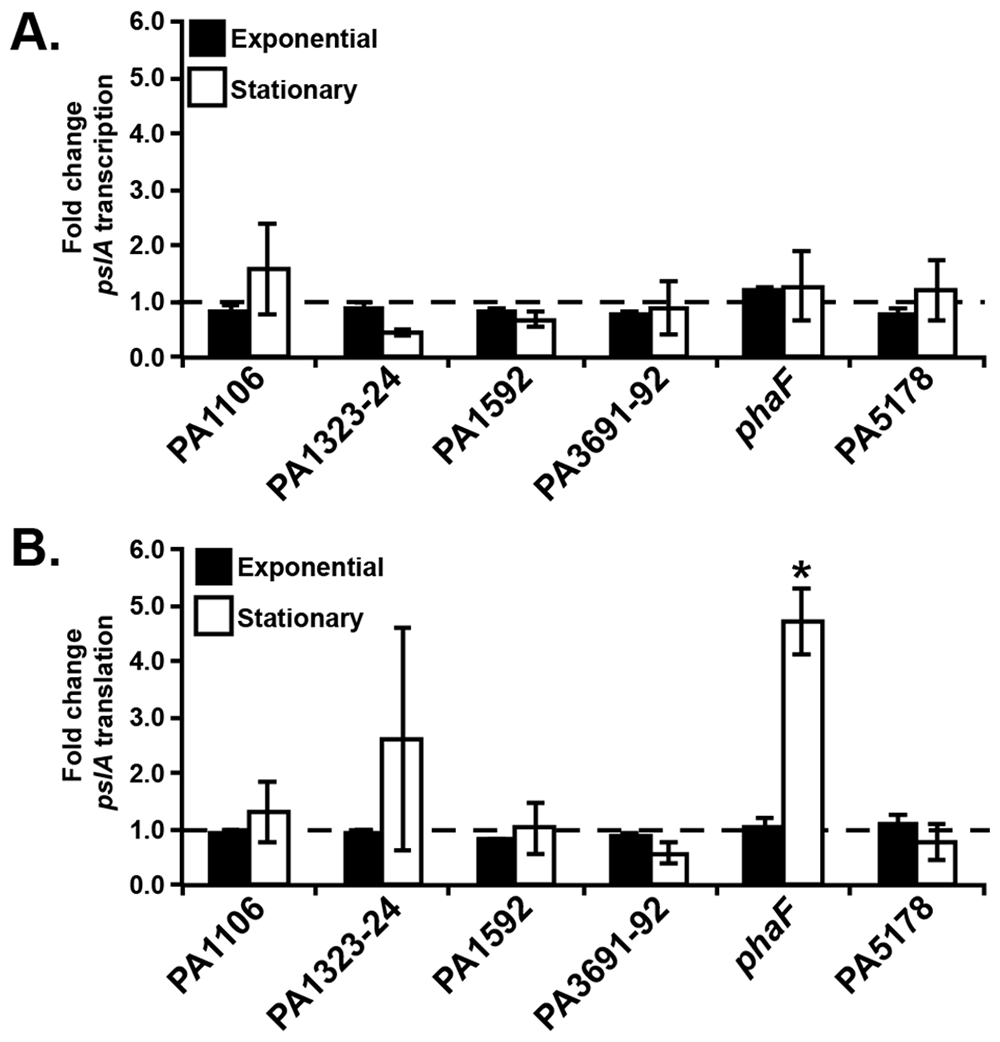
*pslA* transcription and translation in over-expression strains. (A) Fold change in the *pslA* transcriptional response to gene over-expression. (B) Fold change in the *pslA* translational response to gene over-expression. β-galactosidase activity was measured at both exponential (black bars) and stationary phase (white bars). Bars represent fold change in *pslA* transcription or translation compared to the empty vector control strain, PAO1 pJN105 (dashed line). While fold change in *pslA* transcription or translation is reported for clarity, statistics were performed on normalized luminesence values of empty vector control and over-expression strains assayed on the same day (see Materials and Methods). At least 3 biological replicates were performed in duplicate. Error bars represent standard error of the mean, n=3. *, *P* value < 0.05 by Student’s *t* test compared to the empty vector control strain.

Although over-expression of 6 adapted genes/operons enhanced biofilm formation via increased Psl production, it was unclear whether these genes/operons acted within the same or different pathways. *P. aeruginosa* regulates Psl both transcriptionally and post-transcriptionally [17,25], allowing us to test whether these genes/operons affect Psl levels via the same pathway. We constructed strains containing previously characterized chromosomal *pslA*-*lacZ* transcriptional or translational fusions [17] and each over-expression construct. Compared to the empty vector control strain, none of the over-expression strains exhibited altered *pslA* transcription in either exponential or stationary phase (Figure 2A). Similarly, overexpression of PA1106, PA1323-24, PA1592, PA3691-92, and PA5178 did not affect *pslA* translation at either growth stage (Figure 2B). However, overexpression of *phaF* induced an ~5-fold increase in *pslA* translation during stationary phase (Figure 2B). Indeed, microarray analyses verified that *psl* transcription did not change in the *phaF* over-expression strain (Table S2). These data suggest that these genes/operons are affecting Psl levels via at least 2 distinct pathways, as *phaF* is a positive regulator of *psl* translation and the remaining 5 genes/operons affect Psl production via other mechanisms.

### Psl production is enhanced in *P. aeruginosa* chronological clinical isolates

**Figure 3:**
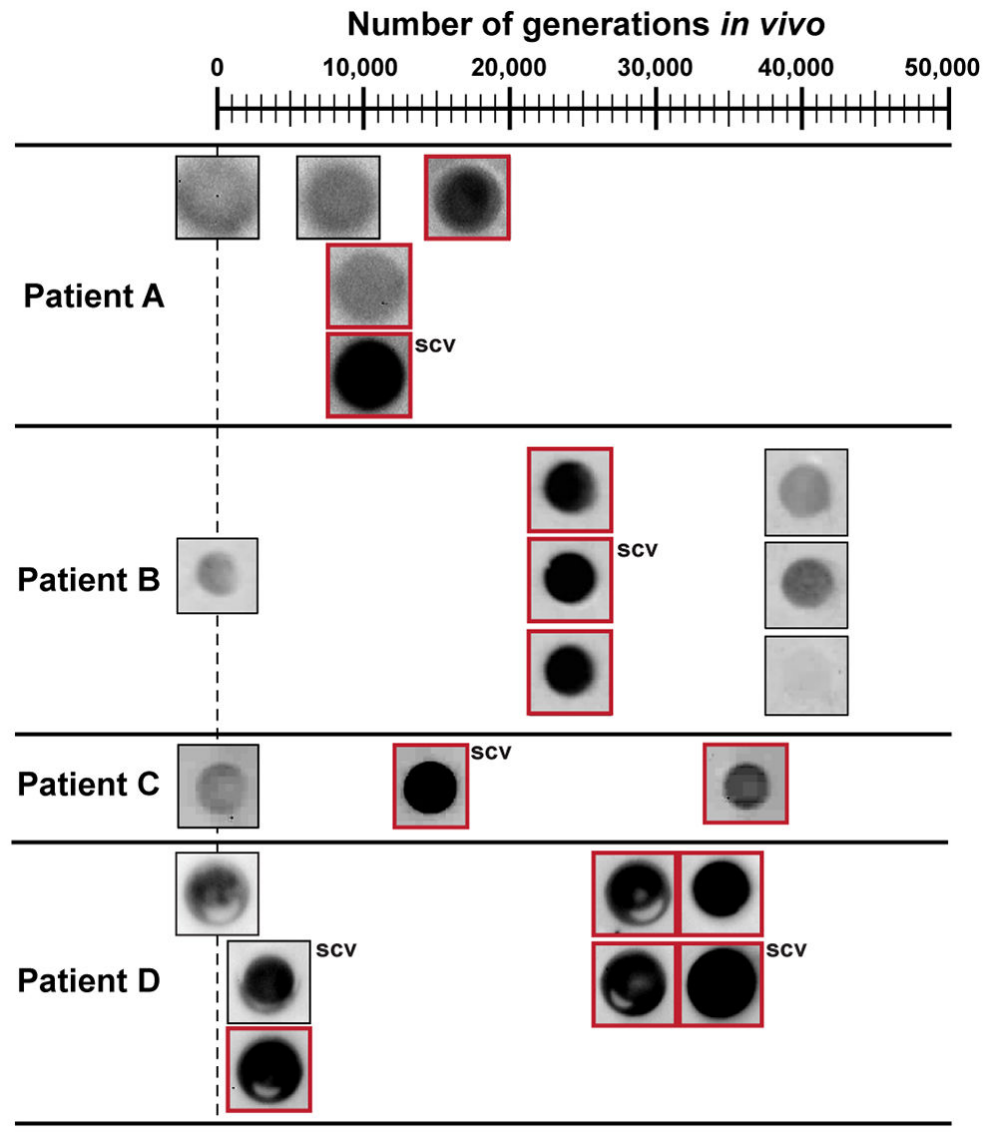
Psl production in clinical isolates from 4 patients with CF. Strains were grown in SCFM for ~16 hours and Psl was extracted from whole cultures. Psl extracts were normalized to equal protein concentrations for each patient and subjected to immunoblot blot analyses as described in the Materials and Methods. Each isolate is centered on its corresponding number of *in* *vivo* generations (Table S3). Isolates at time 0 are the ancestor. At least 3 biological replicates were performed, and red boxes indicate isolates that produced more Psl on average than the ancestor. SCV: small colony variant.

The results described above suggest that over-expression of PA1106, PA1323-24, PA1592, PA3691-92, *phaF*, and PA5178 induce Psl production in strain PAO1. In our previous study, chronic *P. aeruginosa* CF isolates from 3 patients expressed all of these genes at higher levels than their ancestor [10] (Table S3). Therefore, we hypothesized that these chronic isolates would increase Psl production relative to their ancestor strain. To test this hypothesis, we used anti-Psl immunoblotting to compare Psl production in *P. aeruginosa* chronological isolates relative to their ancestors. In 18 chronic *P. aeruginosa* isolates collected from 4 different patients, 13 (~72%) produced more Psl than their corresponding ancestor (Figure 3). This phenotype was expected for at least 5 isolates that showed small colony formation on agar plates (small colony variant; SCV; indicated in Figure 3), but only 4 showed enhanced Psl production compared to the ancestor. Since enhanced Psl production occurred in over 70% of the strains isolated during the first ~40,0000 generations, we propose that enhanced production of Psl is important during adaptation to the CF lung environment.

## Discussion

Since animal models do not manifest chronic *P. aeruginosa* respiratory infections typical of human CF patients, adaptations that improve *in vivo* fitness are difficult to identify. To overcome this challenge, we used an evolutionary approach to identify gene expression traits undergoing natural selection and then characterized the phenotypes these genes impacted. Our studies are similar to *in vitro* microbial evolution experiments that have utilized transcriptomics and genomics to identify beneficial adaptations that arise during standard laboratory growth conditions [26,27]. The strength of these approaches lies in analyzing chronological isolates that have undergone selection, thereby allowing evolution to direct more targeted genetic studies. Here, this powerful approach not only allowed us to identify *P. aeruginosa* traits that are clinically significant, but also illuminated the biological significance of genes with unknown functions.

*P. aeruginosa* chronic CF infections are characterized by an ~40,000 generation adaptation period followed by genetic drift and negative selection [2,3]. Due to the high incidence and association of mucoidy with worsening disease symptoms, many studies have focused on this phenotype [5,9,28]. Indeed, alginate over-production leads to highly structured biofilms with increased antibiotic resistance properties [6]. Here we propose that a non-alginate exopolysaccharide, Psl, is equally important as alginate during adaption to the CF lung. This is based on our data showing that the 6 genes/operons undergoing parallel evolution *in vivo* enhance Psl production, and over 70% of chronic clinical isolates produce more Psl than their ancestor strains during the first ~40,000 generations *in vivo*. Furthermore, recent studies have shown that Psl is essential for biofilm formation even in mucoid strains [29], and small colony variants, characterized as over-producing Psl, can undergo positive selection during chronic infection [30].

While PA1106, PA1323-24, PA1592, PA3691-92, and PA5178 enhance Psl production via an unknown mechanism, *phaF* positively regulates *psl* translation. These data suggest a model where at least 2 distinct pathways are undergoing adaptive evolution resulting in enhanced Psl production. One possible explanation for these findings is that each pathway has additive fitness benefits, thereby allowing selection to act on them separately. Indeed, *in vitro* evolution experiments have shown that multiple genes affecting the same trait can gain mutations that impact fitness [31,32]. While the benefit of enhanced Psl production *in vivo* is unknown, it may be important for antibiotic resistance or for avoiding the host immune response, which has been demonstrated *in vitro* [33,34].

Since the goal of this study was identify *P. aeruginosa* phenotypes undergoing selection *in vivo*, we did not fully investigate the molecular mechanisms of enhanced Psl production in the PAO1 over-expression strains. PA1106, PA1323-24, PA1592, PA3691-92, and PA5178 did not affect either *psl* transcription or translation, while *phaF* enhanced *psl* translation. One explanation for enhanced Psl production in the PA1106, PA1323-24, PA1592, PA3691-92, and PA5178 over-expression strains is that they have high levels of the small signaling molecule bis-(3’,5’)-cyclic-dimeric-guanosine monophosphate (cyclic-di-GMP), which can correlate with increased Psl production without a corresponding increase in *psl* transcription [35]. Additionally, *phaF* over-expression may increase levels of two activators of *psl* translation, the small non-coding RNAs RsmZ and RsmY. However, Northern blot analyses demonstrated that neither RsmZ nor RsmY increased expression in the *phaF* over-expression strain relative to the empty vector control strain (Figure S3). Therefore, we hyopthesize that *phaF* is acting independently of RsmZ and RsmY, and we are currently investigating alternative mechanisms of action.

## Supporting Information

Figure S1 **Biofilm formation of PAO1 and PA14 strains carrying the *phaF* expression construct.** (A) Biofilm formation of PAO1 strains carrying the *phaF* expression construct. (B) Biofilm formation of PA14 strains carrying the *phaF* expression construct. Biofilm formation was tested in the empty vector control strain (black bar) and the *phaF* over-expression strain (white bar) as described in the Materials and Methods. At least 2 biological replicates were performed in octuplicate. *, *P* value < 0.001 by Student’s *t* test compared to the empty vector control strain. Error bars represent standard error of the mean, n ≥ 16.
(TIF)

Figure S2 **Psl anti-sera specificity.** Psl anti-sera specificity was tested on PAO1 control strains grown as described in the Materials and Methods. Psl^-^ (strain WFPA800, a deletion of the *psl* promoter in PAO1). pBAD-*psl* (strain WFPA801, *psl* promoter is replaced with the pBAD promoter in PAO1). PAO1 pJN105 (the wild-type empty vector control strain). See Table S4 for more strain information.
(TIF)

Figure S3 **RsmY and RsmZ expression in the empty vector control (PAO1 pJN105) and *phaF* over-expression strain.** Strains were grown in MOPS buffered minimal media supplemented with 0.5% glucose and 0.5% casamino acids for ~16 hours starting from OD=0.05. 15 μg total RNA was separated on a 10% polyacrylamide-8 M urea gel, transferred to nitrocellulose, and probed for RsmY or RsmZ (see Table S5 for probe sequences). Two biological replicates were performed, and a representative is shown. PAO1 pJN105: empty vector control strain; PAO1 *phaF*: phaF over-expression strain.
(TIF)

Table S1 **Expression of the 15 up-regulated genes in ancestors from 3 CF patients (A1, B1, and C1) compared to PAO1.**
(DOCX)

Table S2 **Expression of the *phaF* over-expression strain compared to the empty vector control strain.**
(DOCX)

Table S3 **Clinical isolates analyzed in this study.**
(DOCX)

Table S4 **Strains and plasmids used in this study.**
(DOCX)

Table S5 **Primers and probes used in this study.**
(DOCX)
