# Supplementary figures and images for: Pseudomonas aeruginosa Enhances Production of a Non-Alginate Exopolysaccharide during Long-Term Colonization of the Cystic Fibrosis Lung
Source: PLoS One. 2013 Dec 6;8(12):e82621. doi: 10.1371/journal.pone.0082621 (PMC3855792; doi:10.1371/journal.pone.0082621)

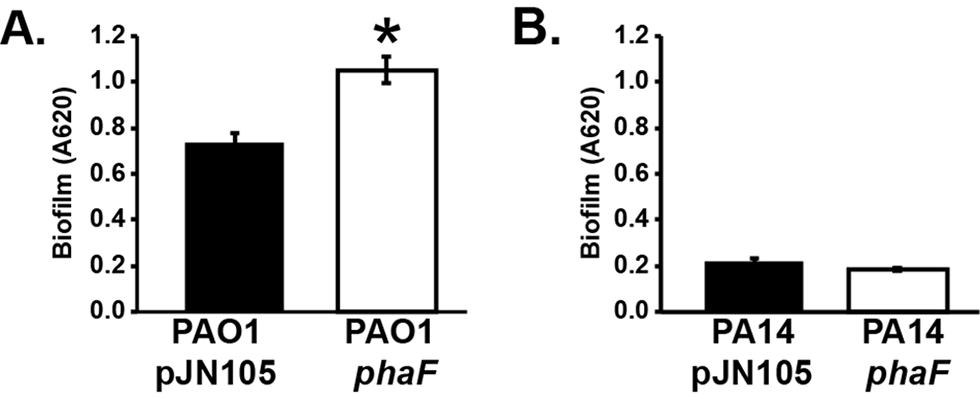

Supplement: Figure S1 — Biofilm formation of PAO1 and PA14 strains carrying the phaF expression construct. (A) Biofilm formation of PAO1 strains carrying the phaF expression construct. (B) Biofilm formation of PA14 strains carrying the phaF expression construct. Biofilm formation was tested in the empty vector control strain (black bar) and the phaF over-expression strain (white bar) as described in the Materials and Methods. At least 2 biological replicates were performed in octuplicate. *, P value < 0.001 by Student’s t test compared to the empty vector control strain. Error bars represent standard error of the mean, n ≥ 16. (TIF) [file pone.0082621.s001.tif]

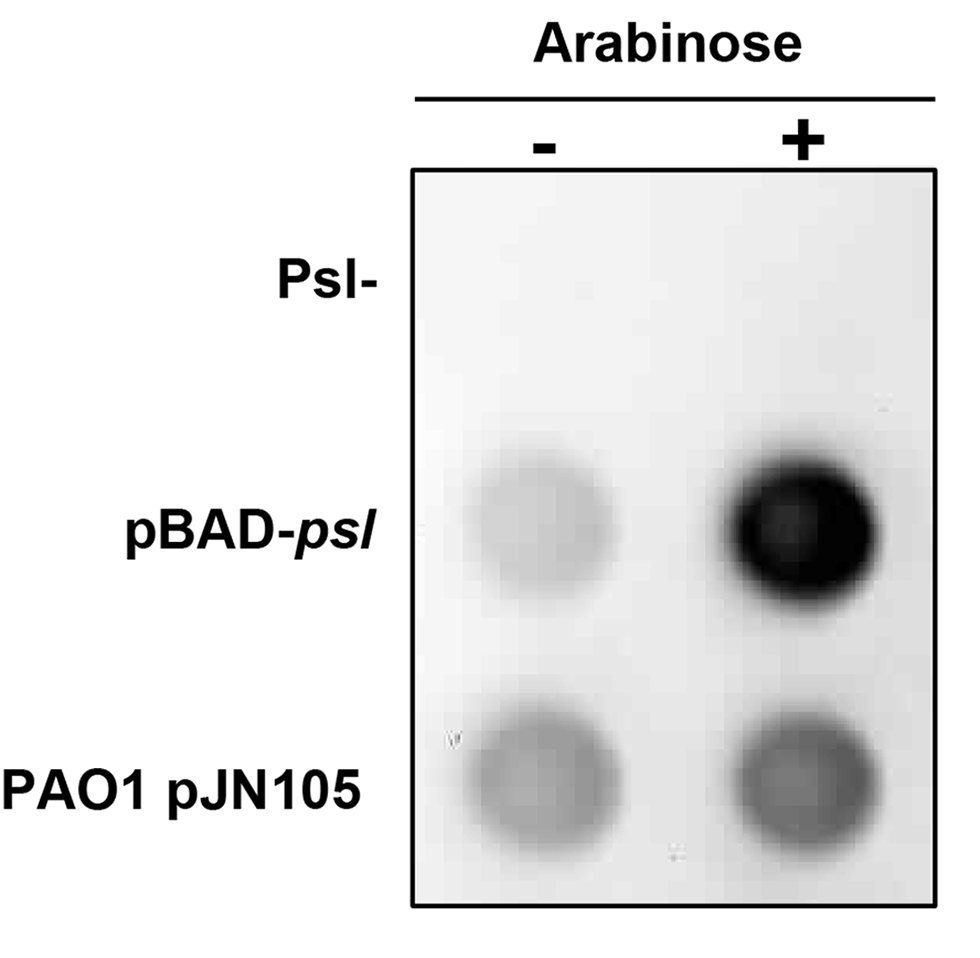

Supplement: Figure S2 — Psl anti-sera specificity. Psl anti-sera specificity was tested on PAO1 control strains grown as described in the Materials and Methods. Psl- (strain WFPA800, a deletion of the psl promoter in PAO1). pBAD-psl (strain WFPA801, psl promoter is replaced with the pBAD promoter in PAO1). PAO1 pJN105 (the wild-type empty vector control strain). See Table S4 for more strain information. (TIF) [file pone.0082621.s002.tif]

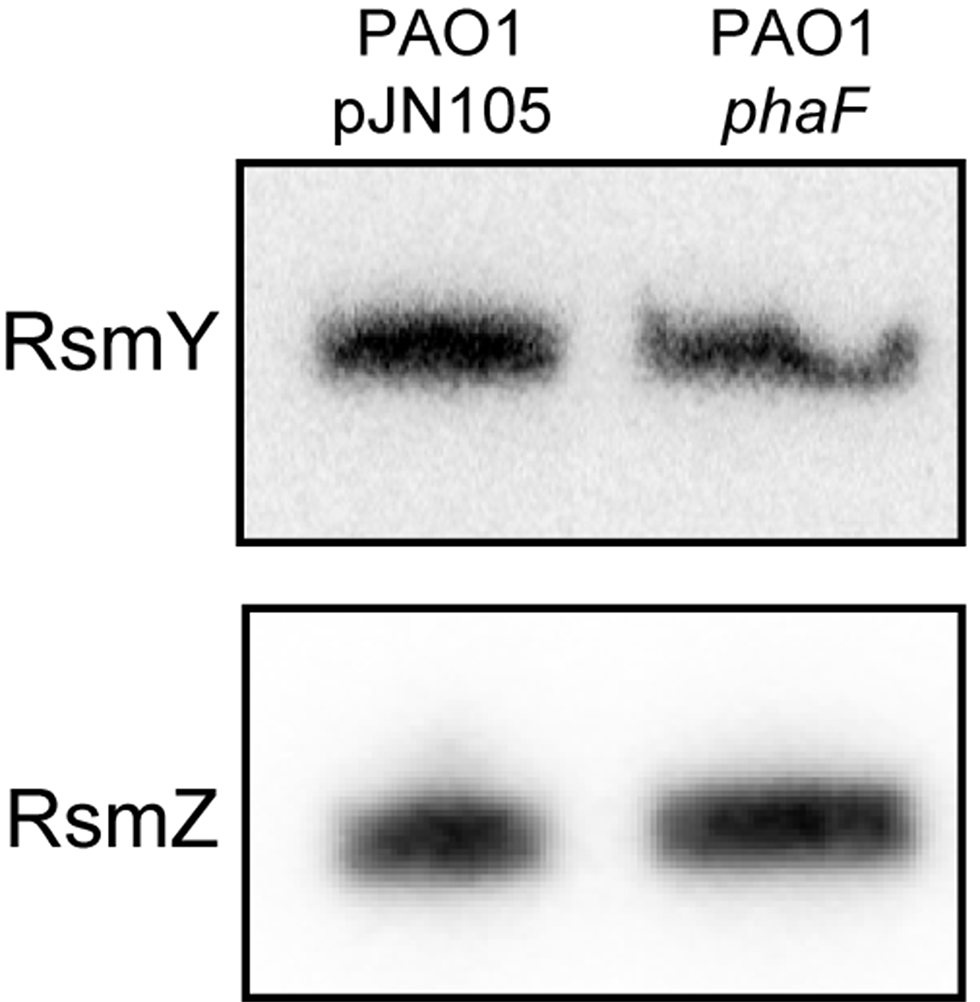

Supplement: Figure S3 — RsmY and RsmZ expression in the empty vector control (PAO1 pJN105) and phaF over-expression strain. Strains were grown in MOPS buffered minimal media supplemented with 0.5% glucose and 0.5% casamino acids for ~16 hours starting from OD=0.05. 15 μg total RNA was separated on a 10% polyacrylamide-8 M urea gel, transferred to nitrocellulose, and probed for RsmY or RsmZ (see Table S5 for probe sequences). Two biological replicates were performed, and a representative is shown. PAO1 pJN105: empty vector control strain; PAO1 phaF: phaF over-expression strain. (TIF) [file pone.0082621.s003.tif]
