# Supplementary material for: Pseudomonas aeruginosa Enhances Production of a Non-Alginate Exopolysaccharide during Long-Term Colonization of the Cystic Fibrosis Lung
Source: PLoS One. 2013 Dec 6;8(12):e82621. doi: 10.1371/journal.pone.0082621 (PMC3855792; doi:10.1371/journal.pone.0082621)
Supplement: Table S1 — Expression of the 15 up-regulated genes in ancestors from 3 CF patients (A1, B1, and C1) compared to PAO1. (DOCX) [file pone.0082621.s004.docx]

**Table S1.** Expression of the 15 up-regulated genes in ancestors from 3 CF patients (A1, B1, and C1) compared to PAO1.

| **Gene^a^** | **A1 (2773C)**^b^ | **B1 (1913C)**^b^ | **C1 (2159M)**^b^ |
| --- | --- | --- | --- |
| PA1048 | NC | NC | NC |
| PA1106 | NC | NC | NC |
| PA1323 | NC | NC | NC |
| PA1324 | NC | NC | NC |
| PA1471 | NC | NC | NC |
| *acnA* | NC | NC | NC |
| PA1592 | NC | NC | NC |
| PA2485 | NC | NC | NC |
| PA2486 | -26 | -23 | -12 |
| PA2778 | NC | NC | NC |
| PA2779 | NC | NC | -4 |
| PA3040 | NC | NC | NC |
| PA3041 | NC | NC | NC |
| PA3042 | NC | NC | NC |
| PA3691 | NC | NC | -5 |
| PA3692 | NC | NC | NC |
| PA4875 | NC | NC | NC |
| PA4876 | NC | NC | NC |
| PA4880 | NC | NC | NC |
| *phaF* | NC | NC | NC |
| PA5178 | NC | NC | NC |

^a^From www.pseudomonas.com

^b^Fold change in gene expression of the ancestor compared to PAO1. Genes were considered differentially expressed if they exhibited > 2-fold change and FDR < 0.05; n=2. NC (no change) indicates that the gene was not differentially expressed between the ancestor *P. aeruginosa* CF strains and PAO1.
