# Supplementary material for: Pseudomonas aeruginosa Enhances Production of a Non-Alginate Exopolysaccharide during Long-Term Colonization of the Cystic Fibrosis Lung
Source: PLoS One. 2013 Dec 6;8(12):e82621. doi: 10.1371/journal.pone.0082621 (PMC3855792; doi:10.1371/journal.pone.0082621)
Supplement: Table S2 — Expression of the phaF over-expression strain compared to the empty vector control strain. (DOCX) [file pone.0082621.s005.docx]

**Table S2.** Gene expression in the *phaF* over-expression strain compared to the empty vector control strain.

| Gene/function^a^ | Number of Genes | Fold change^b^ |
| --- | --- | --- |
| *phaF* | 1 | 3.8 |
| *pslA-O* (Psl biosynthesis) | 15 | NC |

^a^From www.pseudomonas.com

^b^Fold change in the *phaF* over-expression strain compared to the empty vector control strain. Genes were considered differentially expressed if they exhibited > 2-fold change and FDR < 0.05; n=2. NC (no change) indicates that the gene(s) was not differentially expressed.
