# Supplementary material for: Pseudomonas aeruginosa Enhances Production of a Non-Alginate Exopolysaccharide during Long-Term Colonization of the Cystic Fibrosis Lung
Source: PLoS One. 2013 Dec 6;8(12):e82621. doi: 10.1371/journal.pone.0082621 (PMC3855792; doi:10.1371/journal.pone.0082621)
Supplement: Table S3 — Clinical isolates analyzed in this study. (DOCX) [file pone.0082621.s006.docx]

**Table S3.** Clinical isolates analyzed in this study.

| **Patient** | **Label** | **Sample ID** | **Isolation Date** | **Days *in vivo*** | **Colony morphology^a^** | **RAPD type^b^** | **Generations**  ***in vivo***^c^ | **Reference** |
| --- | --- | --- | --- | --- | --- | --- | --- | --- |
| A | A1 | C2773C | 3/26/88 | 0 | Classic | A027 | 0 | Footnote^d^ |
| A | A2 | C3470C | 10/17/89 | 570 | Classic | A027 | 8,208 | Footnote^d^ |
| A | A3.1 | C3639M | 2/27/90 | 703 | Mucoid | A027 | 10,123 | Footnote^d^ |
| A | A3.2 | C3640D | 2/27/90 | 703 | Dwarf/SCV | A027 | 10,123 | Footnote^d^ |
| A | A4 | C4278M | 7/3/91 | 1194 | Mucoid | A027 | 17,194 | Footnote^d^ |
| B | B1 | C1913C | 10/18/86 | 0 | Classic | A055 | 0 | Footnote^d^ |
| B | B2.1 | C4218C | 5/14/91 | 1669 | Classic | A055 | 24,034 | Footnote^d^ |
| B | B2.2 | C4219D | 5/14/91 | 1669 | Dwarf/SCV | A055 | 24,034 | Footnote^d^ |
| B | B2.3 | C4220M | 5/14/91 | 1669 | Mucoid | A055 | 24,034 | Footnote^d^ |
| B | B3.1 | C5912M | 6/8/94 | 2790 | Mucoid | A055 | 40,176 | Footnote^d^ |
| B | B3.2 | C5913C | 6/8/94 | 2790 | Classic | A055 | 40,176 | Footnote^d^ |
| B | B3.3 | C5914M | 6/8/94 | 2790 | Mucoid | A055 | 40,176 | Footnote^d^ |
| C | Cb1 | C2159M | 1/19/87 | 0 | Mucoid | A097 | 0 | Footnote^d^ |
| C | Cb2 | C3488D | 11/9/89 | 1025 | Dwarf/SCV | A097 | 14,760 | Footnote^d^ |
| C | Cb3 | C5623M | 1/7/94 | 2545 | Mucoid | A097 | 36,648 | Footnote^d^ |
| D | D1 | C3881C | 8/28/90 | 0 | Classic | A181 | 0 | This study |
| D | D2.1 | C4197D | 5/7/91 | 252 | Dwarf/SCV | A181 | 3,629 | This study |
| D | D2.2 | C4198C | 5/7/91 | 252 | Classic | A181 | 3,629 | This study |
| D | D3.1 | C6926M | 2/6/96 | 1988 | Mucoid | A181 | 28,627 | This study |
| D | D3.2 | C6927C | 2/6/96 | 1988 | Classic | A181 | 28,627 | This study |
| D | D4.1 | C7514E | 3/18/97 | 2394 | Entire | A181 | 34,474 | This study |
| D | D4.2 | C7515D | 3/18/97 | 2394 | Dwarf/SCV | A181 | 34,474 | This study |

^a^SCV: small colony variant on blood agar

^b^Random Amplification of Polymorphic DNA; demonstrates clonality of isolates (Mahenthiralingam E., Campbell M. E., Foster J., Lam J. S., and D. P. Speert. J. Clin. Microbiol. 34(5):1129-1135, 1996).

^c^As determined by a 100 minute *in vivo* generation time (Yang L., *et al.* J. Bacteriol. 190(8):2767-2776, 2008).

^d^Huse H. K.*, et al.* mBio 1(4), 2010.
