# Supplementary material for: Pseudomonas aeruginosa Enhances Production of a Non-Alginate Exopolysaccharide during Long-Term Colonization of the Cystic Fibrosis Lung
Source: PLoS One. 2013 Dec 6;8(12):e82621. doi: 10.1371/journal.pone.0082621 (PMC3855792; doi:10.1371/journal.pone.0082621)
Supplement: Table S4 — Strains and plasmids used in this study. (DOCX) [file pone.0082621.s007.docx]

**Table S4.** Strains and plasmids used in this study

| Strain or plasmid | Genotype | Reference |
| --- | --- | --- |
|  | | |
| pJN105  PA1048 | *araC*-p*BAD* cloned in pBBR1MCS-5; Gm^r^  PA1048 cloned in pJN105 | foonote^a^  This study |
| PA1106  PA1323-24  PA1471  *acnA*  PA1592  PA2485-86  PA2779-78  PA3040-42  PA3691-92  PA4875-76  PA4880  *phaF*  PA5178  pEX18Gm  pEX18*pslA*  mini-CTX-  *lacZ*::P*_pslA_* TR0  mini-CTX-  *lacZ* EB::P*_pslA_* TR0 | PA1106 cloned in pJN105  PA1323 and PA1324 cloned in pJN105  PA1471 cloned in pJN105  *acnA* cloned in pJN105  PA1592 cloned in pJN105  PA2485 and PA2486 cloned in pJN105  PA2779 and PA2778 cloned in pJN105  PA3040, PA341, and PA3042 cloned in pJN105  PA3691 and PA3692 cloned in pJN105  PA4875 and PA4876 cloned in pJN105  PA4880 cloned in pJN105  *phaF* cloned in pJN105  PA5178 cloned in pJN105  Gene replacement vector, Gm^r^  *pslA* deletion vector, Gm^r^  full length *psl*-*lacZ* transcriptional fusion  full length *psl*-*lacZ* translational fusion | This study  This study  This study  This study  This study  This study  This study  This study  This study  This study  This study  This study  This study  foonote^b^  This study  foonote^c^  foonote^c^ |
|  |  |  |
| *E. coli* |  |  |
| DH5α | *endA1 hsdR17 supE44 thi-1 recA1* Δ*(lacZYA-argF)*  *U169, deoR* [Φ80d*lac* Δ*(lacZ)M15*] |  |
| XL1Blue | *endA1 gyrA96(nal^R^) thi-1 recA1 relA1 lac glnV44*  *F'[ ::Tn10 proAB^+^ lacI^q^ Δ(lacZ)M15] hsdR17(r_K_^-^ m_K_^+^)* | Stratagene |
|  |  |  |
| *P. aeruginosa* |  |  |
| PA14  PA14 pJN105  PA14 *phaF* | Wild-type  PA14 carrying pJN105  PA14 carrying *phaF* | foonote^d^  This study  This study |
| PAO1  PAO1 pJN105  PAO1 *phaF*  PAO1 PA1048  PAO1 PA1106  PAO1 PA1323-24  PAO1 PA1471  PAO1 *acnA*  PAO1 PA1592  PAO1 PA2485-86  PAO1 PA2779-78  PAO1 PA3040-42  PAO1 PA3691-92  PAO1 PA4875-76  PAO1 PA4880  PAO1 PA5178  WFPA800  WFPA800 pJN105  WFPA801  Δ*pslA*  Δ*pslA* pJN105  Δ*pslA* PA1106  Δ*pslA* PA1323-24  Δ*pslA* PA1592  Δ*pslA* PA3691-92  Δ*pslA* *phaF*  Δ*pslA* PA5178  PAO1 mini-CTX-  *lacZ*::P*_pslA_* TR0  PAO1 mini-CTX-  *lacZ* EB::P*_pslA_* TR0  PAO1 mini-CTX-  *lacZ*::P*_pslA_* TR0  pJN105  PAO1 mini-CTX-  *lacZ*::P*_pslA_* TR0  *phaF*-pJN105  PAO1 mini-CTX-  *lacZ* EB::P*_pslA_* TR0  pJN105  PAO1 mini-CTX-  *lacZ* EB::P*_pslA_* TR0  *phaF*-pJN105 | Wild-type  Wild-type PAO1 carrying pJN105  Wild-type PAO1 carrying *phaF*  Wild-type PAO1 carrying PA1048  Wild-type PAO1 carrying PA1106  Wild-type PAO1 carrying PA1323-24  Wild-type PAO1 carrying PA1471  Wild-type PAO1 carrying *acnA*  Wild-type PAO1 carrying PA1592  Wild-type PAO1 carrying PA2485-86  Wild-type PAO1 carrying PA2779-78  Wild-type PAO1 carrying PA3040-42  Wild-type PAO1 carrying PA3691-92  Wild-type PAO1 carrying PA4875-76  Wild-type PAO1 carrying PA4880  Wild-type PAO1 carrying PA5178  *psl* operon promoter deletion mutant in PAO1  WFPA800 carrying pJN105  *psl*-inducible strain, Δ*psl*/P_BAD_-*psl*  clean deletion of *pslA* in PAO1  Δ*pslA* carrying pJN105  Δ*pslA* carrying PA1106  Δ*pslA* carrying PA1323-24  Δ*pslA* carrying PA1592  Δ*pslA* carrying PA3691-92  Δ*pslA* carrying *phaF*  Δ*pslA* carrying PA5178  PAO1 with chromosomal *pslA*-*lacZ*  transcriptional fusion  PAO1 with chromosomal *pslA*-*lacZ*  translational fusion  PAO1 with chromosomal *pslA*-*lacZ*  transcriptional fusion carrying pJN105  PAO1 with chromosomal *pslA*-*lacZ*  transcriptional fusion carrying *phaF*  PAO1 with chromosomal *psl*-*lacZ*  translational fusion carrying pJN105  PAO1 with chromosomal *pslA*-*lacZ*  translational fusion carrying | foonote^e^  This study  This study  This study  This study  This study  This study  This study  This study  This study  This study  This study  This study  This study  This study  This study  foonote^f^  This study  foonote^f^  This study  This study  This study  This study  This study  This study  This study  This study  This study  This study |

^a^Newman J. R. and C. Fuqua. Gene 227(2):197-203, 1999.

^b^Hoang T. T., Karkhoff-Schweizer R. R., Kutchma A. J., and H. P. Schweizer. Gene 212(1):77-86, 1998.

^c^Irie Y.*, et al.*, Mol. Microbiol. 78(1):158-172, 2010.

^d^Liberati N. T.*, et al.*, Proc. Natl. Acad. Sci. U.S.A. 103(8):2833-2838, 2006.

^e^Holloway B. W., Krishnapillai V., and A. F. Morgan. Microbiol. Rev. 43(1):73-102, 1979.

^f^Ma L., Jackson K. D., Landry R. M., Parsek M. R., and D. J. Wozniak. J. Bacteriol. 188(23):8213-8221, 2006.
