# Supplementary material for: Pseudomonas aeruginosa Enhances Production of a Non-Alginate Exopolysaccharide during Long-Term Colonization of the Cystic Fibrosis Lung
Source: PLoS One. 2013 Dec 6;8(12):e82621. doi: 10.1371/journal.pone.0082621 (PMC3855792; doi:10.1371/journal.pone.0082621)
Supplement: Table S5 — Primers and probes used in this study. (DOCX) [file pone.0082621.s008.docx]

**Table S5.** Primers used in this study.

| **Use/primer/probe** | **Sequence^a^** |
| --- | --- |
| Expression constructs in pJN105 | |
| PA1048-F  PA1048-R  PA1106-F | CGGAATTCTCACTTCCAACGGACCCCGAC  GACTAGTTCAGTTGCCCTGGGCCAGGGACA  CGGAATTCCCGCATTACCAGGGGAGAACC |
| PA1106-R  PA1323-24-F  PA1323-24-R  PA1471-F  PA1471-R  *acnA*-F  *acnA*-R  PA1592-F  PA1592-R  PA2485-86-F  PA2485-86-R  PA2779-78-F  PA2779-78-R  PA3040-42-F  PA3040-42-R  PA3691-92-F  PA3691-92-R  PA4875-76-F  PA4875-76-R  PA4880-F  PA4880-R  *phaF*-F  *phaF*-R  PA5178-F  PA5178-R  Deletion of *pslA*  *pslA* EcoRI-F  *pslA* internal-R  *pslA* internal-F  *pslA* KpnI-R  Northern blot analyses  RsmZ probe | GACTAGTTCAGAAGAACGGACGCGACGC  CGGAATTCCATTTTTCAGGGAGCCTCTTC  GACTAGTTCAGCGGGTCAGCAGCACCTT  CGGAATTCTGCGGAGGCATCCCTCAAATG  GACTAGTTCAGCGGTCGTTCTTGGTCGAG  CGGAATTCGCCGTGAGGAAATCAGAAATG  GACTAGTTCAGAGCATGCTGCGCAGCAC  CGGAATTCCCGCACATGGAGGCTAAACG  GACTAGTCTTATTGCTGGTTCTGTTGC  CGGAATTCGAGAAAAAAACACGTCATATGG  GACTAGTTCAGTCGTCGATGCAGCTTTC  CGGAATTCTTTCACGCAGAGGTCATTCCC  GACTAGTTCAGCGGGGCGGTGCGCCAAC  TCCCCGGGGACCAGAGGAGCTCGTCATGC  GCTCTAGATTCAACGGCCTTTCTGCTTGC  CGGAATTCCCCTGTCAGAGGAAATTTCCC  GACTAGTTCAGCCGCTGACGGAGGAGCG  CGGAATTCTTCGCCATCGGCCCGGGGACC  GACTAGTTCAGGGGCGGGCGTCGCGCTC  CGGAATTCCAAGGAGAAAACCGATGACC  GACTAGTTCAGGCGAGGTCGTCGAGGATG  CCGGAATTCGAACCCAATAAGGAGAGCAGG  GACTAGTTCAGCCCTGGCTGCTCGGCG  CGGAATTCAAAGGGAGAACCAAAGATGGG  GACTAGTTCACTCGGGAATGCGCAGGAC  GGAATTCCTGAAGATGCAGCAGCGCTGGGG  CGGCGTTCATCAGTAGACTTCCTTCTGCCGATCACGGGCAGTCCATT  AATGGACTGCCCGTGATCGGCAGAAGGAAGTCTACTGATGAACGCCG  GGGGTACCCGACGATGATCAGGTCGTGCACG  TCATCCTGATGAATCGCCTCCCTGG |
| RsmY probe | CTACGCCACCATCCATGGTGGATTC |

^a^Restriction endonuclease recognition sequences are underlined.
